# Complete mitochondrial genome sequence of lettuce pathogenic fungus, *Fusarium oxysporum* f. sp. *lactucae* 09-002

**DOI:** 10.1080/23802359.2019.1667902

**Published:** 2019-10-09

**Authors:** Jongsun Park, Woochan Kwon, Jung-Bun Kim, Mi-Jeong Park, Tae-Sung Kim

**Affiliations:** a Infoboss Co., Ltd., Seoul, Korea;; b InfoBoss Research Center, Seoul, Korea;; c Department of Agriculture and Life Sciences, Korea National Open University, Seoul, Korea;; d Horticultural and Herbal Crop Environment Division, National Institute of Horticultural and Herbal Science, Rural Development Administration, Wanju, Korea;

**Keywords:** *Fusarium oxysporum* f. sp. lactuace, mitochondrial genome, *Fusarium commune*, Ascomycota

## Abstract

*Fusarium oxysporum* is a famous plant pathogenic filamentous fungus. Here, we report the complete mitochondrial genome sequence of *F. oxysporum* f. sp. *lactucae* isolated from the lettuce field in Suwon area, Korea. Total length of the mitochondrial genome is 45,020 bp and it encodes 42 genes (15 protein-coding genes, two rRNAs, and 25 tRNAs). Nucleotide sequence of coding region takes over 32.7%, and overall GC content is 32.4%. Phylogenetic tree presented that *F. oxysporum* f. sp. *lactucae* 09-002 was clustered with *Fusarium commune* not like another *F. oxysporum* mitochondrial genomes, requiring further analyses.

*Fusarium oxysporum* is an ascomycete and soilborne fungus in every type of soils all over world (Alabouvette and Couteaudier 1992). It is a famous plant pathogenic filamentous fungus which can infect many plant species including crops (Fravel et al. 2003). More than 120 *formae speciales* and races have been classified in *F. oxysporum* (Armstrong 1981). At least 62 mitogenome sequences of *F. oxysporum* have been completed and compared (Pantou et al. 2008; Fourie et al. 2013; Brankovics et al. 2017), presenting diverse features of its mitochondrial genomes as other fungal mitogenomes are (Joardar et al. 2012; Xu et al. 2018; Chen et al. 2019; Park, Kwon, Huang, et al. 2019; Park, Kwon, Zhu, Mageswari, Heo, Han, et al. 2019; Park, Kwon, Zhu, Mageswari, Heo, Kim, et al. 2019). *Fusarium oxysporum* f. sp. *lactucae* 09-002 isolated from wilted lettuce in Suwon area, South Korea (37.28036 N, 127.00870E), was identified based on translation elongation factor 1a gene (Samson et al. 2004). Here, we completed its mitochondrial genome as a first mitogenome in *F. oxysporum* f. sp. *lactuace*.

The hyphae of *F. oxysporum* were collected from samples taken from Horticultural and Herbal Crop Environment Division (*F. oxysporum*, f. sp. *lactucae* 09-002) and its DNA was extracted by using a HiGene™ Genomic DNA Prep Kit (BIOFACT, Korea). Raw data generated by HiSeq4000 were subject to *de novo* assembly done by Velvet 1.2.10 (Zerbino and Birney 2008), gap filling with SOAPGapCloser 1.12 (Zhao et al. 2011), and base confirmation with BWA 0.7.17 and SAMtools 1.9 (Li et al. 2009; Li 2013). Geneious R11 11.0.5 (Biomatters Ltd, Auckland, New Zealand) was used to annotate its mitogenome by comparing with that of *Fusarium commune* strain JCM11502 (NC_036106; Brankovics et al. 2017).

Length of *F. oxysporum* f. sp. *lactucae* 09-002 mitogenome (Genbank accession is MN259515) is 45,020 bp, which is eighth shortest mitogenome among sixty-four mitogenomes (from 34,477 bp to 53,639 bp). It encodes 42 genes consisting of 15 protein-coding genes (PCGs), two rRNAs, and 25 tRNAs, which is similar to those of another *F. oxysporum*. Nucleotide sequence of coding region takes over 32.7%. Overall GC content of this mitochondrial genome is 32.4%, which is the highest GC ratio among 62 mitochondrial genomes of *F. oxysporium*.

Sequence alignment of conserved PCGs extracted from sixty-two *F. oxysporum* mitogenomes, two *F. commune*, and one *F. graminearum* (Al-Reedy et al. 2012) as an outgroup was conducted by MAFFT 7.388 (Katoh and Standley 2013). The neighbor joining and maximum likelihood phylogenetic trees were constructed using MEGA X (Kumar et al. 2018) with 10,000 and 1,000 bootstrap repeats, respectively. Phylogenetic tree presents that our mitogenome is clearly clustered with two mitogenome of *F.commune* (Figure 1). In addition, elongation factor 1a sequence of both species are similar to each other, causing confusion of species identification. Since our strain was isolated from the wilted lettuce, we made a conclusion that it was *F. oxysporium* rather than *F. commune*, which would be confirmed later using more available mitochondrial genomes of *F. oxysporium* and *F. commune* in the near future.

**Figure 1.**
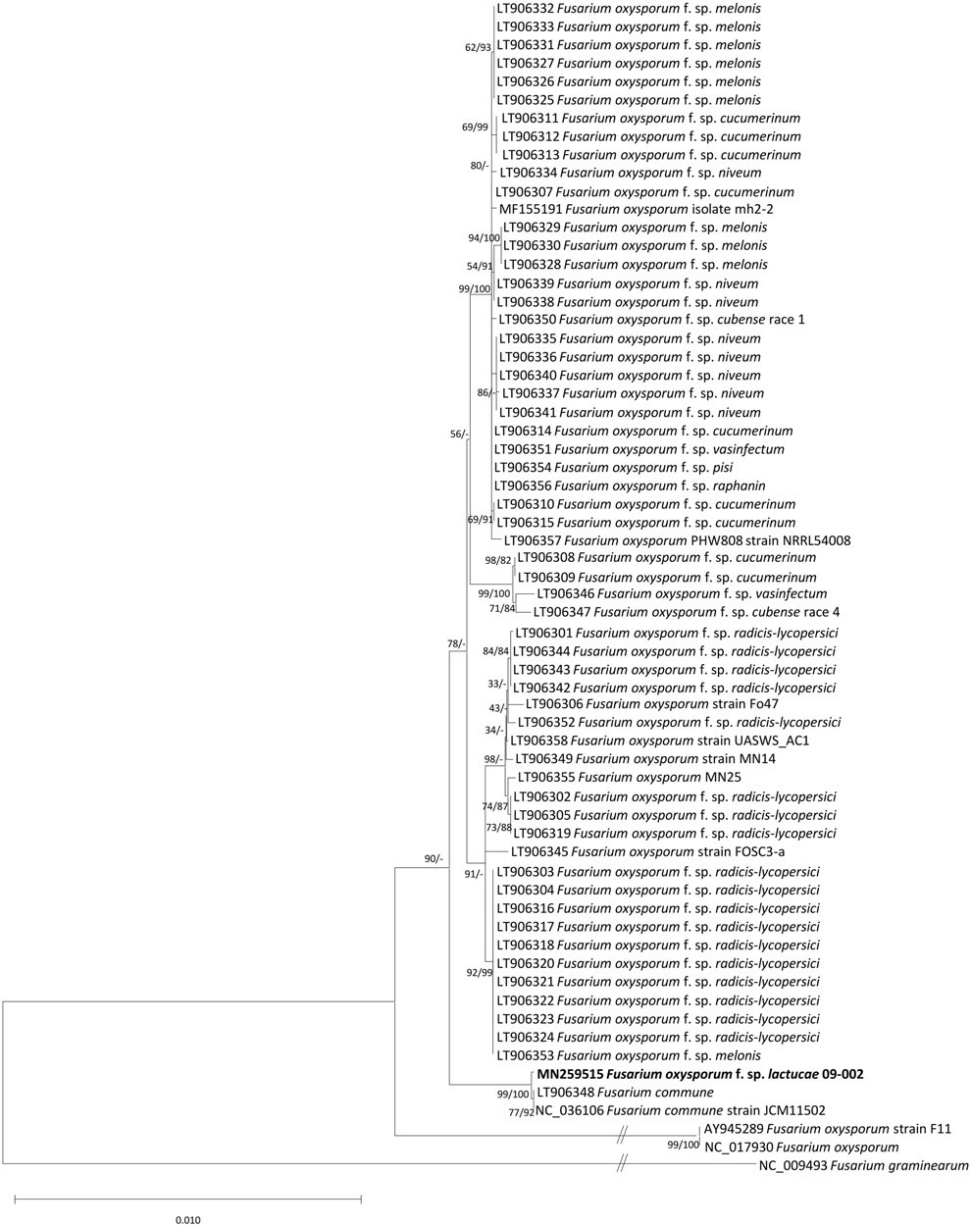
Neighbor joining and maximumlikelihood phylogenetic trees (bootstrap repeat is 10,000 and 1,000, respectively) of 64 *Fusarium* mitochondrial genomes: *F. oxysporum* f. sp. *lactucae* (MN259515 in this study), *F. oxysporum* (NC_017930, AY874423, AY945289, MF155191, LT906306, LT906349, LT906355, LT906358, LT906357, and LT906345), *F. oxysporum* f. sp. *melonis* (LT906328, LT906329, LT906330, LT906325, LT906326, LT906327, LT906331, LT906332, LT906333, and LT906353), *F. oxysporum* f. sp. *pisi* (LT906354), *F. oxysporum* f. sp. *vasinfectum* (LT906351), *F. oxysporum* f. sp. *raphanin* (LT906356), *F. oxysporum* f. sp. *cubense* race 1 (LT906350), *F. oxysporum* f. sp. *cubense* race 4 (LT906347), *F. oxysporum* f. sp. *niveum* (LT906334, LT906338, LT906335, LT906336, LT906340, LT906341, LT906339, and LT906337), *F. oxysporum* f. sp. *cucumerinum* (LT906315, LT906314, LT906310, LT906307, LT906308, LT906309, LT906311, LT906312, and LT906313), *F. oxysporum* f. sp. *radicis-lycopersici* (LT906352, LT906342, LT906343, LT906344, LT906302, LT906305, LT906319, LT906301, LT906303, LT906304, LT906316, LT906317, LT906318, LT906320, LT906321, LT906322, LT906323, and LT906324), *F. oxysporum* f. sp. *vasinfectum* (LT906346), *F. commune* (LT906348 and NC_036106), and *F. graminearum* (NC_009493) as an outgroup. Phylogenetic tree was drawn based on neighborjoining tree. Numbers on branches indicate bootstrap values of neighbor joining and maximum likelihood phylogenetic trees, respectively.
